# Malaria vaccines and their potential role in the elimination of malaria

**DOI:** 10.1186/1475-2875-7-S1-S10

**Published:** 2008-12-11

**Authors:** Geoffrey A Targett, Brian M Greenwood

**Affiliations:** 1 Department of Infectious and Tropical Diseases, London School of Hygiene & Tropical Medicine, Keppel Street, London WC1E 7HT, UK

## Abstract

Research on malaria vaccines is currently directed primarily towards the development of vaccines that prevent clinical malaria. Malaria elimination, now being considered seriously in some epidemiological situations, requires a different vaccine strategy, since success will depend on killing all parasites in the community in order to stop transmission completely.

The feature of the life-cycles of human malarias that presents the greatest challenge to an elimination programme is the persistence of parasites as asymptomatic infections. These are an important source from which transmission to mosquitoes can occur. Consequently, an elimination strategy requires a community-based approach covering all individuals and not just those who are susceptible to clinical malaria.

The progress that has been made in development of candidate malaria vaccines is reviewed. It is unlikely that many of these will have the efficacy required for complete elimination of parasites, though they may have an important role to play as part of future integrated control programmes. Vaccines for elimination must have a high level of efficacy in order to stop transmission to mosquitoes. This might be achieved with some pre-erythrocytic stage candidate vaccines or by targeting the sexual stages directly with transmission-blocking vaccines. An expanded malaria vaccine programme with such objectives is now a priority.

## Background

Development of a malaria vaccine has been difficult. Greatly expanded investment in malaria vaccine research and development in recent years has resulted in the identification of a substantial number of vaccine candidates that are now in clinical trials or in the late stages of preclinical development. Now the malaria vaccine community is faced with a new challenge. Do the vaccine development plans developed several years ago, when the main target of malaria vaccine development was reduction in the burden of clinical malaria, fit with the new and ambitious aim of achieving malaria elimination. Here the current situation with respect to malaria control, the particular challenges elimination strategies present, and the progress being made in vaccine development are considered. An assessment is made of what vaccines are needed and how they could be used most effectively as part of a malaria elimination programme.

The much quoted figures for malaria deaths and clinical cases – around 1 million deaths and 300–500 million clinical cases per annum, are still the best estimates available. The majority of these deaths are due to *Plasmodium falciparum *malaria and occur in sub-Saharan Africa [1]. The importance of *Plasmodium vivax *infection, in particular in South-East Asia, and the severity of some infections caused by this malaria parasite have been underestimated but are now receiving more attention [2].

There are, however, encouraging recent reports that show that a very significant improvement in the malaria situation is possible using existing control tools. Effective malaria control in high transmission areas seemed a remote possibility, even just a few years ago, but, with the substantial increase in political commitment and financial investment in control measures over the past 5–6 years, some dramatic results have been obtained. Some of the reported successes have occurred in countries or regions where malaria transmission was already low [3] but, in other cases, a significant downward trend has been achieved in places where transmission is stable; Zanzibar [4], Eritrea [5], The Gambia [6] and Kenya [7,8] are good examples.

These successes have involved scaling up of existing control measures, notably treatment with artimisinin-based combination therapy (ACT) or other effective drug combinations, insecticide-treated nets (ITNs) and, increasingly, a return to insecticide-residual spraying (IRS). There has also been increased use of intermittent preventive treatment in pregnancy [9], and this approach to malaria control is being explored in infants and older children. A high level of commitment to the discovery of new drugs and insecticides is essential to ensure that these gains are not lost when the drugs and insecticides in current use lose their effectiveness.

Effective malaria control is defined as a reduction in cases of clinical malaria and mortality to a level at which malaria ceases to be a major problem. The malaria parasite still persists in the community, country or region and, if the control measures are not sustained, there is every likelihood that transmission and numbers of cases will increase rapidly again. However, the somewhat surprising impact of scaling-up the use of existing control measures has prompted the call, first by the Bill and Melinda Gates Foundation, quickly endorsed by WHO, and then by the Roll Back Malaria partnership, for malaria elimination to become the new goal. This has a very different and far more challenging aim of stopping transmission completely within a defined region, so that the only cases of malaria that occur are through importation from outside the region [10]. Elimination by this definition can be achieved only by killing all of the parasites within the target population.

It is clear that, despite the successes achieved by scaling-up use of existing tools, additional or alternative strategies will be needed if malaria elimination is to be achieved – a possible exception being some island situations [11].

### Persistence of infection

The focus of enhanced research and malaria control has, understandably, been primarily on *P. falciparum *malaria, given the mortality and severity of disease associated with this species. However, there is increasing recognition that the risk of infection and the burden of disease due to *P. vivax *malaria is substantial and, although of limited importance in sub-Saharan Africa, this parasite is often the dominant one in the other major endemic regions of the world. Frequently, *P. vivax *and *P. falciparum *occur sympatrically and elimination programmes, in such cases, must take account of the different challenges presented by the two species, especially in terms of persistence of infection, and the complex interactions between the species. This complex balance may be disturbed by vaccination against either *P. falciparum *or *P. vivax *in areas where both parasites are prevalent. Eliminating *P. falciparum *but not *P. vivax *would be a step forward, but if this was all that was achieved could damage the reputation of a malaria elimination programme.

Malaria elimination means stopping infection. This may be achieved if the measures directed against asexual parasites are fully effective, or by targeting sexual stages directly with drugs or vaccines.

### Transmission of *P. falciparum*

In areas of high transmission, asexual parasite densities are highest in young children, and it is in this age group that microscopic detection of gametocytes is most common. Both asexual blood stage and gametocyte densities then decline with age, though the patterns of decline are somewhat different [12]. Epidemiological studies show, however, that transmission of *P. falciparum *is as dependent on the parasites not detected by routine blood screening as on those that are readily seen. The cumulative evidence for the importance of low-grade asymptomatic infections as a reservoir for infection of mosquitoes is strong. In areas of highly seasonal malaria, where there is often a long dry season in which little or no transmission occurs, persisting very low gametocytaemias are the source from which transmission occurs at the onset of the subsequent rainy season [13]. In areas where the endemicity of malaria allows acquired immunity to develop, there are many asymptomatic individuals, particularly adults, who are an important source of infection for mosquitoes [14,15].

Detection of gametocytes using molecular techniques, such as reverse transcriptase polymerase chain reaction (RT-PCR) [13] and quantitative nucleic acid sequence-based amplification (QT-NASBA) has shown that gametocytaemias can persist at sub-microscopic levels for months and that the prevalence of gametocytaemic individuals is much higher than was assumed from blood film examinations [16]. Of particular relevance to consideration of malaria elimination, Shekalaghe *et al *[17], in a study of parasite prevalence in an area of low, seasonal transmission in Tanzania, showed that, while microscopically the parasite rates were low (1.9% asexual parasites, 0.4% gametocytes), QT-NASBA revealed much higher prevalence rates (32.5% asexual parasites and 15.0% gametocytes).

These observations indicate the need to adopt a community-based approach to elimination; any selective interventions used should be based more on the focality of malaria [18], rather than on particular groups especially at risk from the clinical consequences of malaria infection, such as young children or pregnant women. Those at low risk clinically may still be important transmitters of infection.

### Transmission of *P. vivax*

A key factor in the persistence of *P. vivax *is the fundamental difference in the life cycle shown by *P. vivax *and *Plasmodium ovale*, compared with *P-. falciparum *and *Plasmodium. malariae*, namely the occurrence of dormant hypnozoites in the liver, from which relapse infections can emerge, weeks, months or years after infection.

There is some evidence from use of PCR techniques, that, as for *P. falciparum*, the number of *P. vivax *infections in an affected community is generally significantly underestimated [19]. Though the pattern of gametocyte production in *P. vivax *is different from that of *P. falciparum*, low grade asymptomatic infections are equally likely to give rise to infectious gametocytes.

### Progress with vaccine development

The challenges set to vaccine developers [20] by those who drew up the malaria vaccine road map are first, by 2015, to produce a licensed vaccine that has a protective efficacy of more than 50% against severe disease and death from malaria which lasts longer than one year. Secondly, by 2025, to develop a vaccine with a protective efficacy greater than 80% against clinical disease and death that lasts longer than four years.

These targets focus on the prevention of clinical disease, especially its severe and life-threatening form, valid objectives for vaccines that are to be introduced into areas of medium or high transmission. A vaccine that conformed to the 2015 objective, providing protection to half of those vaccinated, would be valuable as part of an integrated control programme alongside vector control and chemo-preventative measures. That level of efficacy would not justify its use alone as an alternative to other means of malaria control.

The expanding programme of experimental vaccine-related research has two broad but overlapping approaches. One is to achieve a much needed better understanding of the nature of protective immune mechanisms against malaria, thus providing a basis for rational vaccine design [21]. The other approach is more empirical and involves issues of design and presentation as vaccines of antigens that have been recognised for a long time and which are known to have an important role in the parasite's life cycle.

### Pre-erythrocytic stage vaccines

The primary objective of pre-erythrocytic stage vaccines is provision of a level of protection that prevents any invasion of the blood and hence any clinical malaria. This has been achieved readily in experimental malarias by vaccination with radiation-attenuated sporozoites; this formed the starting point for the extensive investigations into pre-erythrocytic vaccines. Complete protection was also achieved in humans against *P. falciparum *and *P. vivax *by exposing them to the bites of mosquitoes that had been irradiated to attenuate their sporozoites. However, this delivery procedure was totally impractical and the research emphasis shifted to the synthetic design and genetic-engineering of sub-unit vaccines [22].

There has, however, been a renewal of interest in the development of attenuated-sporozoite vaccines. Given the high level of sterile protection these whole organism vaccines can induce, this is a very welcome development. Stephen Hoffman established and directs Sanaria Inc. specifically to produce radiation-attenuated sporozoites of *P. falciparum *from infected mosquitoes in sufficient quantity and in a way that meets the regulatory standards required for their use as a vaccine [23]. This has been achieved and phase I/2a clinical trials will begin shortly using irradiation-attenuated sporozoites delivered by intradermal or subcutaneous injection.

Genetic attenuation of sporozoites is also being investigated. Mueller *et al *[24] produced sporozoites from *Plasmodium berghei *deficient in the vis3 gene. These gave complete protection when used for vaccination. Sporozoites lacking 6-cysteine secretory proteins required for parasitophorous vacuole formation are equally effective vaccines [25]. The immune responses of importance following vaccination with attenuated sporozoites are CD8+T cell-mediated with production of interferon gamma [26-28].

Synthetic and genetically-engineered sub-unit vaccines have generally been based on the two surface proteins, circumsporozoite protein (CSP) and thrombospondin-related anonymous protein (TRAP) involved in sporozoite motility and invasion of liver cells. Most of the phase 2 trials of vaccines based on these antigens, using a variety of vaccine constructs, have given poor results and it is perhaps surprising that the RTS, S vaccine candidate has proved to be much more promising. This vaccine is a hybrid molecule expressed in yeast, that consists of the tandem repeat tetra-peptide (R) and C-terminal T-cell epitope containing (T) regions of CSP fused to the hepatitis B surface antigen (S), plus unfused S antigen. The adjuvant ASO2, which consists of an oil in water emulsion containing immunostimulants monophosphoryl lipid A and QS-21, a fraction of *Quillaia saponaria*, is an essential component of the vaccine. Variants of ASO2 have been tested successfully [29], and an alternative adjuvant, ASO1, where a liposomal formulation replaces the oil in water emulsion, has given very encouraging results [30,31].

The first challenge trials with RTS, S/ASO2 gave impressive, but short-lived, protection in naïve adults [32]. Similarly, in a trial in Gambian adults, there was greater than 70% protection against parasitaemia in the nine weeks post-vaccination, but the immunity waned rapidly [33]. The most comprehensive study of RTS, S/AS02 has been in children in Mozambique, who have been followed up for two years. Over this period, there was 30% protection against clinical malaria and close to 50% protection against severe malaria [34]. Most recently, in a small-scale trial in infants designed primarily to test safety and immunogenicity, RTS, S/AS02D had a vaccine efficacy of 65.9% against new infections in the six months of follow-up [35]. A series of phase 2 studies preparatory to a large phase 3 trial and potential licensure of this vaccine are in progress.

RTS, S is several years ahead of any other vaccine in terms of assessment of its efficacy in clinical trials. Trials of other pre-erythrocytic stage vaccines, based on CSP, TRAP and other liver stage antigens, several of which have used viral vector delivery systems, have shown some initial promise, but are not sufficiently advanced or effective to be considered yet for evaluation in phase 3 trials [36,37].

### Asexual blood-stage candidate vaccines

A range of blood stage antigens have progressed to phase 1 and phase 2 trials. Most are molecules that have been identified as being involved in the process of invasion of erythrocytes by merozoites [38]. Promising results from studies with rodent malarias are proving difficult to transfer to human infections [39-43], though some early encouraging results with MSP-3 antigen have been reported [44]. The expectation is that such candidate vaccines might give protection against disease, but not against infection. This was the case with one phase 2 clinical trial of a vaccine containing MSP-1, MSP-2 and RESA antigens which reduced parasite density, but not prevalence of infection [45]. It was also strain-specific in its effect and the polymorphism of these antigens, coupled with the variability in invasion pathways *P. falciparum *can adopt [46,47] are a severe challenge to the design of this kind of vaccine. Blocking invasion of reticulocytes by *P. vivax *merozoites might be a more hopeful strategy, given that the Duffy antigen is thought to be the obligatory receptor on the erythrocyte surface [48] yet, even here an alternative invasion strategy has been proposed [49].

*Plasmodium falciparum*-infected erythrocytes express highly variable parasite molecules on the red blood cell surface. Naturally-acquired immunity involves variant-specific responses to these antigens and the very complexity of these responses may limit the potential of these antigens as vaccine candidates. However, this variability might be exploitable for specifically targeted vaccines. The *P. falciparum*-infected erythrocytes that sequester in the placenta of pregnant women have a very selected sub-set of variant surface antigens, notably one coded VAR2CSA, through which they bind to chondroitin sulphate A (CSA) in the placenta. This opens the possibility of designing a vaccine that would be beneficial to pregnant women [50], but which would probably not affect the other variants that circulate and sequester elsewhere using different receptor-ligand interactions. Another postulated approach to vaccination, much less studied, is to block parasite molecules that mediate disease by inducing pro-inflammatory responses. Glycosylphosphotidyl-inositol (GPI) is strongly implicated [51], but any vaccine effect would alleviate symptoms of disease without affecting infection. The immunity induced by such a vaccine might prevent some of the severe complications of malaria mediated by cytokine-induced response to infection. Use of vaccines of this kind, however, has the potential disadvantage of damping down the early clinical features of malaria, such as fever, which could delay the time before a patient sought treatment whilst still allowing parasite multiplication to occur.

### Transmission-blocking vaccines

The concept of a malaria vaccine that could provide an effective immune response when the antibodies induced had been ingested by blood-feeding mosquitoes, was developed thirty years ago [52]. The target antigens of the passively transferred antibodies that blocked transmission were shown to be sexual-stage specific surface molecules (Pfs 48/45 and Pfs 230 in *P. falciparum*), that are involved in the process of fertilization of macrogametocytes by microgametes. Subsequently, other antigens (P25 and P28 proteins), that are uniquely expressed in the mosquito by zygotes and ookinetes (i.e. after fertilization), were shown to be equally good for induction of transmission-blocking immunity (TBI). The end result in each case is to prevent sporogonic development in the vector.

Experimental studies with animal models have shown that it is possible to induce a highly effective TBI [22,53]. The most extensive studies have focused on Pfs 48/45, Pfs 230, Pfs 25, Pfs 28 of *P. falciparum *and orthologues in other *Plasmodium *species, but more potential vaccine candidates have been identified [54].

The gamete surface molecules (48/45 and 230) are also expressed in gametocytes circulating in the blood. This has made it possible to study the nature and duration of naturally-acquired sexual-stage specific immunity, and has contributed importantly to an understanding of the epidemiology of gametocytes in comparison with that of the much more comprehensively studied asexual blood stages [12]. The P25 and P28 proteins are not expressed in gametocytes and hence there is no natural infection-related immunity to them. However, the mRNA that encodes these proteins is measurable in gametocytes and is used as the basis of highly sensitive means of detecting gametocytes [16].

The transmission-blocking activity of sera from vaccinated animals or humans, or from individuals naturally infected, has mostly been assessed by an *ex vivo *assay, during which mosquitoes feed through a membrane on blood containing gametocytes and a serum under test. Comparison with controls of the numbers of mosquitoes that become infected, and the number of oocysts they carry, gives a measure of the potency of the serum under test.

Experimentally, sera from rabbits, monkeys and mice vaccinated with vaccine candidates Pfs25 from *P. falciparum *and Pvs25 from *P. vivax *contained antibodies with transmission-blocking activity. The antibody levels measured by ELISA correlated with both oocyst reduction and the number of mosquitoes that failed to become infected [55]; antibody levels persisted at a high level for months after a second or third injection in mice [56]. Phase 1 human vaccine trials were also effective [57], though not yet at the level that will be required and can be achieved experimentally.

Alternatives to the membrane-feeding assay are also being assessed for evaluation of transmission-blocking activity. Transmission of the transgenic *P. berghei *expressing the P25 antigens of either *P. falciparum *[58] or *P. vivax *[59] was blocked by antibodies obtained from animal vaccination and phase 1 clinical trials.

Though Pfs48/45 has been clearly shown to induce antibodies that prevent fertilization and correlate with transmission-blocking activity, the conformational nature of the epitopes, and the cysteine-rich nature of the protein has made production of a correctly folded recombinant molecule difficult. Encouragingly, production of a stable, properly folded C terminal portion of the molecule that induces transmission-blocking antibodies has recently been described [60,61].

### Use of vaccines in an elimination programme

Since elimination of malaria requires complete removal of all parasites, the focus of measures used to accomplish this goal is quite different from that for disease control. Whether the measures used for parasite elimination involve drugs [9] or vaccines, or a combination of the two, what is required are tools that prevent production of gametocytes or that render them non-infective to mosquitoes.

The ideal vaccine would be one that induces complete sterilizing immunity or which is fully effective at blocking transmission. Nothing approaching induction of a sterile immunity has been shown so far and it seems unlikely that this will be achieved with sub-unit vaccines. This does not rule out the use of such vaccines as part of an integrated approach to malaria-elimination, but they are unlikely to induce an anti-parasite immunity of sufficient efficacy to eliminate all parasites. It remains to be seen whether attenuated sporozoites, which, in small-scale studies with a demanding and unusable vaccination regime, did give full protection, will be as effective in the trials now beginning.

The transmission-blocking vaccines currently being tested induce good, but certainly not complete, transmission-blocking immunity. Since the membrane-feeding and the transgenic rodent malaria assays allow sera from clinical trials to be tested for efficacy, it is possible to set up a series of small-scale phase 1/2a trials with different vaccine constructs and vaccination regimes. It should then be possible to make speedy progress towards improving vaccine efficacy and selecting the best vaccine for development. There is an urgency to do this.

There are biological and clinical features of infection that need to be taken into account in designing elimination measures that include vaccines. These are listed in Table 1 and, in particular, involve the interaction between the acquired immune response and the gametocyte infectious reservoir. As discussed earlier in this review, the gametocyte reservoir is grossly underestimated when assessed by conventional means, even in areas of low transmission. The same is true of asexual parasitaemias. In areas of stable transmission, numbers of gametocytes decrease with age more rapidly than do numbers of asexual parasites, but the proportion of individuals who are gametocytaemic is actually higher at lower transmission intensities [12]. An increase in gametocyte prevalence has also been seen following control studies [62]. For this and other reasons adult carriage becomes as important as that of children in terms of the source of infection for mosquitoes.

**Table 1:** Acquired immunity, persistence of gametocytes, and transmission.

1.	Acquired immunity to asexual blood stages increases with exposure but allows the persistence of low level parasitaemias from which gametocytes develop.
2.	Anti-parasitic immunity will persist after interruption of transmission and may allow the occurrence of asymptomatic infections and hence gametocytaemias for a number of years.

3.	Transmission-blocking immunity to gametocytes develops rapidly then wanes so that adults carrying small numbers of gametocytes are less likely to have antibodies that render them non-infective.

4.	Low levels of transmission-blocking antibodies have been shown to enhance transmission.

5.	The gametocyte reservoir is much larger than that determined by blood film examination even in areas where transmission is low. Transmission of infection can occur from individuals with very low numbers of gametocytes.

6.	Gametocyte infectivity is broadly related to gametocyte density but small numbers of infectious gametocytes in adults are as important in maintaining transmission as larger numbers of gametocytes in children susceptible to clinical attacks of malaria.

7.	A vaccine for elimination of malaria must ensure everyone susceptible to malaria infection is protected completely or that all gametocytes are made non-infective.

Naturally acquired immune responses to the sexual stages of the malaria parasite develop rapidly (in individuals with little or no previous exposure to malaria) and have transmission-blocking activity [63]. However, in endemic areas, the ability of sera to reduce transmission decreases in older age groups, corresponding to a reduction in gametocyte numbers [64]. The biological significance of this loss of immunity has been reviewed [12] and further supports evidence that adults with few parasites are nevertheless important as a reservoir of infection. There is evidence too, particularly for *P. vivax*, that a low level of immunity can enhance transmission to mosquitoes [65].

It is frequently stated that, as malaria control and elimination programmes progress, the population no longer has any immunity and becomes highly susceptible to epidemic infections. While this is true to some extent, especially for younger age groups, there is another aspect of immunity to consider. It is well known that those who had immunity to malaria quite rapidly lose their clinical immunity if no longer exposed and may develop a symptomatic infection at low parasite densities on subsequent exposure. However, the anti-parasite immunity they had acquired can persist for many years [66-68]. In other words, there is immunological memory directed against the infection. The relevance of this to malaria elimination is that, for many years, there may be a proportion of the population capable of supporting low grade infections from which gametocytes can be derived.

## Conclusion

Elimination programmes are focused on populations, not individuals, and, optimally, a herd immunity that is sustained and prevents transmission is required. The current vaccine development programmes are largely concerned with disease control and must be sustained, but, from the results obtained so far in clinical trials of the RTS, S vaccine, the impact is not dissimilar to that of ITNs, i.e. there is a greater impact on severe disease than on infection. It is difficult to imagine partially effective asexual blood stage vaccines being very useful for parasite elimination. Given that all age groups can potentially provide a source of infection to mosquitoes, a high proportion of the population will need to be given a vaccine. However, there is much evidence to show that malaria is a highly focal disease and, initially at least, it might be beneficial to vaccinate those at greatest risk of being bitten by vector mosquitoes. Whatever vaccine is employed, pre-erythrocytic or transmission-blocking, its efficacy would need to be very high to achieve elimination [9].

A transmission-blocking vaccine has no direct effect on clinical malaria, but would break the life-cycle between human and malaria. It might be used as a stand-alone vaccine, but, more appropriately, as part of an integrated programme involving drug-treatment and vector control. The concept of a multi-component, multi-stage vaccine [69] with an effect from one component mainly on disease and from another on infection is intuitively appealing though the type of vaccine design required to kill parasites or render gametocytes non-infective to mosquitoes might be quite different from vaccines whose beneficial effect is mainly against disease severity. What is needed is an expanded malaria vaccine programme targeting the particular requirements of malaria elimination.

## Competing interests

The authors declare that they have no competing interests.

## Authors' contributions

GT prepared the first draft of the manuscript. BG and GT determined the content of the review and the reference sources, and agreed the final form of the manuscript.
